# Panorama of the Intracellular Molecular Concert Orchestrated by Actinoporins, Pore-Forming Toxins from Sea Anemones

**DOI:** 10.3390/toxins13080567

**Published:** 2021-08-13

**Authors:** Carlos Alvarez, Carmen Soto, Sheila Cabezas, Javier Alvarado-Mesén, Rady Laborde, Fabiola Pazos, Uris Ros, Ana María Hernández, María Eliana Lanio

**Affiliations:** 1 Centro de Estudio de Proteínas, Facultad de Biología, Universidad de La Habana (UH) and Laboratorio UH-Centro de Inmunología Molecular, Havana CP 11600, Cuba; carmensoto@fbio.uh.cu (C.S.); sheilacabezas@gmail.com (S.C.); javier.alvarado.mesen@una.ac.cr (J.A.-M.); radylq@fbio.uh.cu (R.L.); fpazos@fbio.uh.cu (F.P.); urosquin@uni-koeln.de (U.R.); mlanio@fbio.uh.cu (M.E.L.); 2 Escuela de Ciencias Biológicas, Universidad Nacional, Heredia 40101, Costa Rica; 3 Institute for Genetics and Cologne Excellence Cluster on Cellular Stress Responses in Aging-Associated Diseases (CECAD), University of Cologne, Joseph-Stelzmann-strasse 26, 50931 Cologne, Germany; 4 Immunobiology Division, Molecular Immunology Institute, Center of Molecular Immunology (CIM), Playa, Havana CP 11600, Cuba; maraborys42@gmail.com

**Keywords:** actinoporin, pore-forming proteins, pore-forming toxins, cytolysin, intracellular signaling, cell death

## Abstract

Actinoporins (APs) are soluble pore-forming proteins secreted by sea anemones that experience conformational changes originating in pores in the membranes that can lead to cell death. The processes involved in the binding and pore-formation of members of this protein family have been deeply examined in recent years; however, the intracellular responses to APs are only beginning to be understood. Unlike pore formers of bacterial origin, whose intracellular impact has been studied in more detail, currently, we only have knowledge of a few poorly integrated elements of the APs’ intracellular action. In this review, we present and discuss an updated landscape of the studies aimed at understanding the intracellular pathways triggered in response to APs attack with particular reference to sticholysin II, the most active isoform produced by the Caribbean Sea anemone *Stichodactyla helianthus*. To achieve this, we first describe the major alterations these cytolysins elicit on simpler cells, such as non-nucleated mammalian erythrocytes, and then onto more complex eukaryotic cells, including tumor cells. This understanding has provided the basis for the development of novel applications of sticholysins such as the construction of immunotoxins directed against undesirable cells, such as tumor cells, and the design of a cancer vaccine platform. These are among the most interesting potential uses for the members of this toxin family that have been carried out in our laboratory.

## 1. Introduction

Pore-forming toxins (PFTs) are one of the oldest and most amazing tools employed by living organisms to attack or self-defend. Synthesized as soluble molecules, PFTs experience conformational changes in response to different stimuli that ultimately lead to pore-formation in the target cell membrane. These conducting channels can be either lytic to the target cell, e.g., by creating an osmotic imbalance, or can mediate the translocation of proteins, namely toxins, into the cell cytoplasm. PFTs are commonly categorized into two broad groups depending on the elements of the secondary structure used to span the cell membrane. α-PFTs utilize amphipathic α-helices to cross the bilayer whereas β-PFTs form amphipathic β-barrel pores [1]. Regardless of the mechanism used, all PFTs are characterized by their extraordinary ability to transmute from water-soluble molecules to proteins stably associated with membranes. Some of the best-studied PFTs are three members of the α-PFTs (colicins, AP, and ClyA) and three of the β-PFTs (hemolysins β, aerolysin β, and Cholesterol Dependent Cytolysins) [1]. Their common functional feature is the ability to pierce cell membranes which ultimately leads to cell death. This is achieved through a variety of molecular strategies for self-assembly in the lipid bilayer that are not yet very clear.

All PFTs are synthesized in their soluble form, which associates with the membrane, forming aqueous pores in the target cells. These pores unbalance cellular homeostasis by increasing the non-selective passage of molecules. In any case, pores from different PFTs exhibit a variety of characteristics that could induce different cell death phenotypes [1,2,3]. The injuries of the plasma membrane are profoundly disparate in size and properties, and these properties condition how cells manage their membrane repair mechanisms [4,5]. Holes originated by PFTs in the membrane cause devastating damage to the target cell. In response to this aggression, organisms respond intracellularly by mobilizing complex regulatory and interconnected mechanisms against this injury, including restoration of cell membrane integrity.

Despite the extraordinary diversity of PFTs in terms of origin, structure, or function, they all display a similar mode of action [2,6], with bacterial PFTs being the best-characterized group [1]. Eukaryotic PFTs have been less investigated than their bacterial peers, possibly because of their lesser significance to human health. In this review, we discuss the major alterations that APs, a family of eukaryotic PFTs, elicit in cells. The understanding of the mechanism of pore formation in membranes by APs has provided the basis for the development of their applications as biomedical and biotechnological tools, such as the development of a cancer vaccine platform [7,8].

## 2. Actinoporins Are Potent Toxins Produced by Sea Anemones

Sea anemones (Actiniaria) synthesize different classes of cytolytic polypeptides [9]. One of them, comprising mainly basic ~20 kDa proteins, discovered as lethal hemolysins or cytolysins and inhibited by sphingomyelin, was named actinoporins [10]. These proteins are produced by anemones for defense and attack purposes [11]. Among PFTs, APs have aroused the interest of the scientific community due to their biomedical or biotechnological potential to build immunotoxins [12,13,14], vaccine platforms [7,8], and nanopore-based biosensors [15,16,17]. Fragaceatoxin C (FraC) produced by *Actinia fragacea* [18,19], equinatoxin II (EqTII) from *Actinia equina* [20,21] and sticholysins (Sts), I (StI), and II (StII) purified from *Stichodactyla helianthus* [22,23,24,25] are the most extensively studied toxins in this family. APs exist as isoforms in most sea anemones which exhibit diverse pI, molecular weight, and piercing activity. These diverse isoforms produced by a single species belong to one multigene family. Indeed, they differ in a few amino acids, but several dissimilarities in terms of solubility, hemolytic activity, and interaction with cholesterol have been found. These isotoxin variations within the same species have been hypothesized to contribute to expanding the range of targets the venom can act on as a defensive weapon [26,27,28,29,30,31,32,33].

APs are monomeric, soluble, α-helical PFTs with a molecular mass of around 20 kDa, the majority with a basic pI (>9.0), lacking Cys residues, and a high affinity for sphingomyelin-containing membranes [9].

StI and StII have a molecular weight of ~19 kDa exhibiting elevated sequence similarity (99%) and identity (93%) [34,35]. Sts readily associate to cell and model membranes forming pores in them. Their transmembrane α-helical barrel pores perturb cellular ionic gradients, elicit osmotic swelling, and eventually lead to cell death [36,37]. The pore radius formed by APs has been determined to be of ~1 nm radius [18,37,38,39,40].

**Figure 1:**
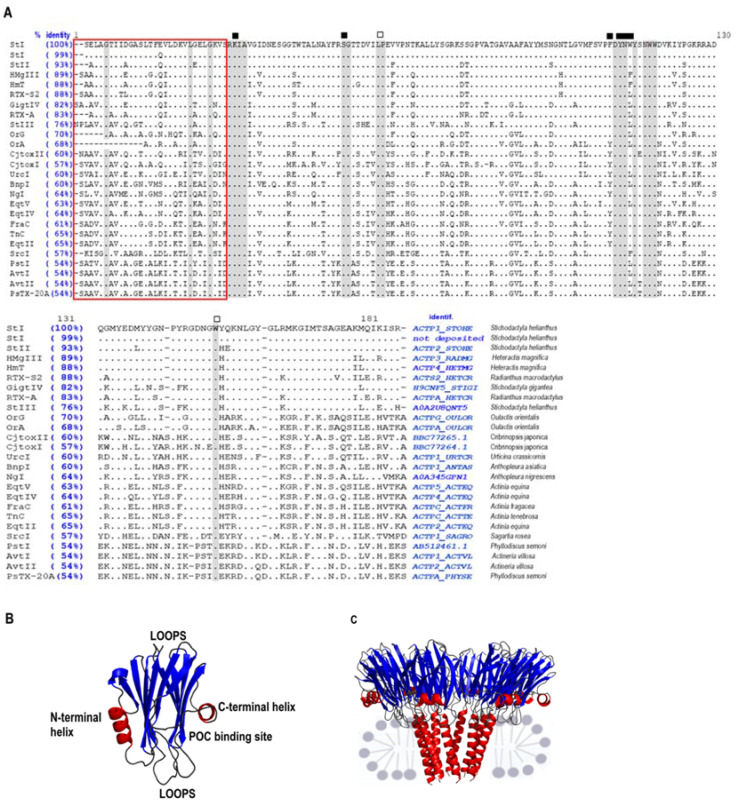
Structural features of APs. (**A**) Multiple sequence alignment of the full-length actinoporin sequences. The amino acid sequences of actinoporins were obtained from the non-redundant protein databases using the NCBI BLAST protein server, BLASTp (https://blast.ncbi.nlm.nih.gov/Blast.cgi, accessed on 2 August 2021), from the National Library of Medicine, USA, and were aligned with that of StI as described [31]. Identical amino acids were identified with dots, spaces with a dash, and substitutions with the corresponding amino acids. The amino-terminal segments (approximately the first 30 amino acids) are enclosed in a red rectangle. Some of the amino acids strictly conserved in the sequences are shaded, and their functions in the protein–protein, protein–lipid interaction, or both, are identified with an open black square, solid black square, and white diamond, respectively, as described [31]. (**B**) Schematic representation of StII 3D structure. StII structure displayed in a ribbon diagram (PDB: 1O72-A) exhibiting common structural features of APs. The red ribbon symbolizes the helixes in blue β-sheets; turns and loops are shown in gray. Furthermore, the POC binding site is shown. The structure was estimated by Pymol Software [45]. (**C**) Representation of a side-view showing the octameric pore of FraC in a lipid bilayer [18].

The three-dimensional (3D) structures in a solution of five APs have been solved: StI [41], StII [25], EqTII [20,21] FraC [18], and FraE [42]. A similar 3D fold has been described for all of them as expected from their high sequence similarity (Figure 1A). Their overall structure is characterized by a stiff central core formed by two β sheets and by two α-helices arranged perpendicularly to each other on both sides of the central β core (Figure 1B). The α-helix located closest to the N-terminal helix is amphipathic, mobile, and flexible and is involved in pore-formation (Figure 1C) [25,41,43]. Additionally, the structure of soluble StII, in a complex with phosphocholine (POC), showed a binding site for the phospholipid headgroup, which is positioned in a protein region with a remarkable abundance of aromatic amino acid residues. The amino acids involved in this binding site are highly conserved in APs, implying that there are common features of lipid recognition by other toxins of the family [44]. The POC binding site is a cleft formed by amino acids of the second α-helix, the β-sheet core, and the array of aromatic amino acids (Figure 1B).

Together, the POC binding site and the aromatic residue cluster form a structural element that is essential for the association of these proteins to membranes, which is termed the interfacial binding site [44]. The structure of FraC was resolved by X-ray crystallography at four steps of its cytolytic mechanism, (1) the water-soluble state, (2) the lipid-bound form, (3) an intermediate complex, and (4) the structured transmembrane pore uncovering novel highlights of the APs permeabilizing process. As a result, the existence of various sites for lipid binding were described [18]; two of them (L2 and L3) were assumed to be early binding sites, analogous to the StIIߣs POC binding site [25]. Besides, locations L4 and L5 were postulated to be sites of small affinity for POC or likely high-affinity binding sites for other lipids bearing headgroups different from POC [18].

### Mechanism of Pore Formation by APs

PFTs recognize the target cell by associating to specific receptors or a complex mixture of them. Sugars, lipids, and proteins have been documented as PFTs receptors. As the first stage of the PFT mechanism, membrane binding fulfills the function of reducing the dimensionality of the molecular diffusion of the toxin protomers from the third dimension to the two-dimensionality of the membrane plane. In this way, the local concentration of the toxin is increased, which is a favorable event for the mandatory oligomerization that follows in the mechanism. Oligomerization may precede or be simultaneous to the exposition of hydrophobic surfaces leading to membrane insertion [1].

The mechanism of pore formation by APs in membranes involves a several stage-mechanism: (i). membrane binding, (ii). oligomerization, detachment and insertion of the N-terminus into the hydrophobic nucleus of the membrane, and (iii). pore assembly [25,41,43,46,47]. The binding of the protomers to the membrane through the aromatic residues and lipid-binding sites was identified as the first stage in this sequence. The resulting membrane-associated structure is similar to that in the solution, implying that no significant conformational changes are involved in this transition [48,49,50]. Upon binding to the membrane, the concomitant association of several AP monomers and the relocation of the N-terminus from the toxin body to the hydrophobic nucleus of the bilayer takes place. The precise sequence of events occurring during the oligomerization step is likely the most disputed issue of the mechanism of pore-formation by APs [3]. One model suggests the partial detachment of the N-terminus from the protein body triggered upon binding to the membrane [49,50]. In this model, the displacement of the N-terminus into the bilayer occurs in an uncoordinated manner, before or simultaneously with the oligomerization process, as has been proposed for EqTII [51] or StI [52]. By using planar lipid membranes, it was shown that upon the binding of StI and insertion of its N-terminus into the membrane, pore assembly occurs by passing through several transient sub-conductance states. These three or four states seem to occur due to the consecutive incorporation of N-terminal α-helices and the headgroup of lipids to the arising pores until a stable oligomeric structure is assembled. Thus, the pore is structured after the consecutive addition of the N-termini of various monomers and lipid molecules [52]. Interestingly, an oligomeric intermediate of FraC has been observed and characterized by cryo-electron microscopy in liposomes of phosphatidylcholine. Even though it has not been confirmed that this intermediate is of relevance for the insertion of APs in liposomes of sphingomyelin/phosphatidylcholine, it may be of importance for the lipid compositions [53]. Recently, it has been postulated that the thermodynamically stable pores formed by Sts in the membrane are at least heptameric, although any higher stoichiometry should not be completely ruled out, given some of the relative uncertainties of these experiments based on Förster Resonance Energy Transfer (FRET) studies. Interestingly, these results seem to make it clear that the existence of oligomers of five or fewer entities is unlikely since they could not be detected by this refined approach [54]. Interestingly, these results are consistent with the stoichiometry of the crystallized FraC pore [18] and with topological studies of StI in membranes by site-directed spin labeling and electron paramagnetic resonance (EPR) spectroscopy. Indeed, EPR studies evinced that StI bound to the membrane displays an oligomeric architecture with a non-homogenous stoichiometry of primarily eight or nine monomers, according to available structural data [55]. When taken together, the experimental evidence converges that the oligomers of membrane-bound Sts, and most likely those of other APs, are formed by oligomers composed of more than seven protomers that aggregate into a thermodynamically stable assembly. Lower order oligomers seem to be formed in the transition to these more stable higher-order structures, as revealed by single-molecule analysis in cells treated with EqTII [56]. Another debate in this area revolves around the existence or not of prepore structures in the APs mechanism of action [18,25,40]. However, an in-depth analysis of the pore assembly kinetics does not support the formation of a stable oligomer prior to the insertion of the α-terminal helix into the lipid bilayer [47,51]. Experimental evidence reveals the importance of dimers as intermediates in the assembly process of pore formation by APs EqTII [56], StII [57,58], and FraC [18].

## 3. Piercing Cells by APs: Cell Death and Survival Mechanisms

The pathways that follow pore-formation by PFTs in the cell membrane may differ depending on the cell type, its physiological conditions, and the nature of the insult. There is great diversity in the type and complexity of cellular responses to APs aggression. These cover a wide spectrum from that of the simplest, non-nucleated cells (for instance, mature mammalian erythrocytes) to the most complex nucleated cells where multiple interconnected mechanisms are orchestrated to give a cellular response. In nucleated cells, lysis may be a late consequence of AP injury, as the mechanisms responsible for compensating for the damage, including cell membrane repair, are unable to respond effectively, and the cell succumbs. In the following section, we will describe the effects promoted by APs when bound to the membrane of target cells. A significant part of these outcomes will be exemplified with the contributions of our laboratory in order to understand the function of StI and StII, two of the most studied APs [22,23]. In addition, we will summarize the approaches and results reported by our laboratory concerning the characterization of injuries inflicted by Sts in nucleated and non-nucleated cells.

### 3.1. The Colloid-Osmotic Shock Causes Cell Death in Non-Nucleated Erythrocytes 

The lytic activity of APs can be assessed directly by testing their ability to form pores in the red blood cell membrane, i.e., by measuring their hemolytic activity. Like most APs, Sts are very effective in causing the lysis of non-nucleated red blood cells from mammalian species, e.g., rats, sheep, rabbits, and humans [59]. The concentration at which 50% of a human erythrocyte suspension (C50, ~5 × 10^6^ cells·mL^−1^) is hemolyzed after exposure to the toxin (30 min) is around 0.1 nM or 0.3 nM for StII and StI, respectively. In most of the red blood cells studied, StII shows greater hemolytic activity than StI [22,45]. Hemolysis was elicited by Sts as well as the rest of APs results from a colloidal osmotic shock prompted by pore-formation in the membrane. Hemolysis can be prevented by the addition to the incubation medium of an osmotic protectant of a sufficiently large size that does not allow access to the cell interior through the pores and thus counterbalances the increase in intracellular osmotic pressure. As for StI and StII, the presence of such large protectants augmented the time t_1/2_ required to reach 50% hemolysis depending on their size [37]. This strategy was employed to determine the pore dimensions originated by Sts in red blood cells and liposomes. Thereby, the pore radius estimates for StI were 1.09 nm when the evaluation was carried out with oligosaccharides and 0.96 nm when determined with polyethylene glycols (PEG), whereas for StII the radius deduced with PEG in red blood cells was 1.05 nm [37]. These studies showed that Sts’s pore has a constant size (~1.1 nm), which is independent of sticholysin concentration and is quite similar in nature and model to membranes, indicating that it assembles in a primarily fixed structure [37].

### 3.2. APs Cytotoxicity on Nucleated Eukaryotic Cells

Similar to what occurs in model membrane systems and erythrocytes, APs are also able to open pores in the membranes of nucleated eukaryotic cells. This perforation causes the death of many different cell types as long as their membranes contain sphingomyelin [60]. However, very few studies have documented the interaction of APs with nucleated cells and their implications for intracellular processes. The first evidence of the cytotoxic activity of a purified hemolytic fraction from *S. helianthus* on human nucleated cells was obtained in cells from myelocytic-, lymphoblastic- and chronic myelogenous leukemia, as well as in peripheral mononuclear cells [61]. Subsequently, the cytotoxic effect of this hemolytic fraction was also demonstrated on two human breast carcinoma lines [62], while the effect of purified StI was firstly examined on human colorectal cancer cells [63]. The cytotoxic activity (C_50_) ranged from 0.3 nM to 6.0 nM in correspondence with values reported for other members of the APs family (0.01–100 nM) [64]. In the same direction, studies carried out with RTX-A, produced by the sea anemone *Radianthus macrodactylus*, showed potent cytotoxicity (C_50_ = 1.0–5.0 nM) against human tumor cell lines, such as HL-60, MDA-MB-231, HeLa, THP-1, and SNU-C4 in agreement with the cytotoxicity values obtained earlier for other members of the APs family [22,65]. In contrast, more recently, a recombinant variant of Hct-S3 (rHct-S3), from the combinatory library of *Heteractis crispa* (synonym *Radianthus macrodactylus*) displayed cytotoxicity against breast MDA-MB-231, colorectal HT-29, and melanoma SK-MEL-28 cancer cells but in the micromolar concentration range. In this case, the AP was purified after a rather complex construction and purification strategy that could have affected the native toxin folding [66]. The cytolytic activity of StII effectively induces a reduction in cell viability in diverse nucleated mammalian cells, as determined by membrane integrity, namely by measuring propidium iodide uptake and lactate dehydrogenase release as resulting from cell lysis following pore formation. The concentration of toxin required to cause the death of about 50% of cells in the assay (C_50_) is in the nanomolar range in correspondence with previous studies with other AP family members in different cell models [12,60,67].

### 3.3. Early Signals following Pore-Formation

The primary response after the interaction of PFTs with cells is the lack of membrane integrity and the subsequent change in the ionic balance of the cytoplasm. The efflux of intracellular K^+^ is an early indicator of the permeability of the cell membrane by a PFT [6]. In the case of Sts, the outflow of internal K^+^ takes place before hemolysis, suggesting that the rate-determining step in the process is not the channel formation. The comparison of erythrocyte K^+^ outflow provoked by similar concentrations of both isoforms showed that StII is more efficient than StI. Interestingly, the time delay between K+ outflow and hemolysis is quite similar for both toxins [68]. A rapid K^+^ outflow and delayed hemoglobin release have also been observed as a consequence of the activity of other APs on erythrocytes [69,70], characteristic of a colloid-osmotic type of cell lysis. The cytotoxic activity of APs may also result from a rapid influx of extracellular Ca^2+^ in favor of its gradient into the cytosol, which can evoke huge crosstalk of uncontrolled processes mediated by this cation [71]. Ca^2+^ seems to penetrate into the cytosol through the pores of StII [72,73], as it has also been shown for the related AP EqTII [71,74]. It has been postulated that Ca^2+^ entering the cell through StII pores appears to activate the flip-flop movement of membrane phospholipids, which may contribute to membrane destabilization and consequently cell death [73].

The pores resulting from the Sts–erythrocyte interaction perturb the ionic gradients resulting from Cl^−^ and Ca^2+^ influx as well as K^+^ outflow from the cells, leading to an increase in cell volume and ultimately cell demise [72,73,75]. In addition, little is known about the intra-cell response at the molecular level and whether or not cells can recover after membrane injury. This is also the case for bacterial PFTs. Interestingly, in this direction, it has been documented that aerolysin and listeriolysin O (LLO) at sublytic concentrations induce a decrease in cytoplasm K^+^ and a concomitant increase of Ca^2+^ [76].

Lytic concentrations or long-lasting exposure to PFTs elicit irreversible plasma membrane perturbation, leading to uncontrolled necrotic cell death [77]. During necrosis induced by PFTs, the cell swells, loses its boundaries, and finally dies, usually by undergoing blebbing which sequesters and sheds the toxin to survive [5,74]. Swelling blebs are globular protuberances that classically form on the cell surface after profound membrane harm. Their function under pathological conditions remains controversial, but it has been argued that they might represent a liquid reservoir to alleviate the increased cellular volume of water and thus may exert a protective action against osmotic cell lysis [78,79,80]. However, at sub-lytic doses, PFTs can promote pyroptosis, necroptosis, or apoptosis, which affects the consequence of an infection in vivo [6,77,81,82,83,84]. Pathogens producing PFTs activate different cell death pathways [84]; however, the mechanisms that underlie the activation of a particular death pathway can vary depending on the cell type or organism. It is not yet clear whether the diverse cell death responses are either favorable for the host through inducing protection, or for pathogens through eliciting infection.

Even at sublytic concentrations, PFTs are toxic as they can still alter cell behavior. The permeabilization of the cell membrane caused by PFTs inevitably increases membrane permeability; however, the permeability to particular molecules to which the cell membrane becomes permeable can differ. Depending on the toxin in question, the resulting channel may allow the passage of only specific ions, such as K^+^ and/or Ca^2+^; or mediate the transport of rather small molecules (e.g., ATP) or larger ones (for instance, proteins) [1]. Most of the studies indicate that APs [85], as with most of the bacterial PFTs [86], seem to prompt fast necrosis in diverse cell types under lytic conditions. Beyond these observations, the molecular mechanisms conducive to cell death caused by APs remain poorly understood. Due to this knowledge gap, efforts have been devoted to identifying putative targets and to uncovering intracellular signal transduction pathways that may be triggered by APs. This understanding is essential for their potential biomedical/biotechnological applications; however, to date it is extremely difficult to arrive at generalizations. Given the experimental evidence, it is most likely that the cellular response to a given toxin relies on the cell type, the intensity of toxin–membrane interactions (i.e., whether it only disturbs membrane organization or installs pores), and on the size stability and relative homogeneity of the lesion. Changes in the intracellular K^+^ content are considered to be the main signal that a perturbation of the plasma membrane barrier has occurred by a PFT [76]. We investigated the cell damage caused by the StII on a eukaryotic cell, and its connection to intracellular K^+^ concentration. This study evinced that toxin action on non-tumor Baby Hamster Kidney cells (BHK) was detectable a few minutes after the addition of the toxin. The appearance of blebs that grew over time was a morphological modification observed in the vast majority of StII-treated cells [5]. However, membrane repair was not effective at high toxin concentrations. In contrast, at sublytic concentrations, a relevant loss of cell cytosol K^+^ due to membrane damage occurs during the first hour of StII interaction with cells. Although, in the following hours, the intracellular K^+^ concentration increased, which could be an indication of cell recovery [5,6]. Surprisingly, it was found that the recovery of the membrane after the action of the StII damage occurred on a similar time scale to that of the bacterial LLO injury despite the fact that these two PFTs form pores differing greatly in size. Moreover, membrane recovery after StII addition takes place in a completely different time frame than that observed for aerolysin, another bacterial PFT that also produces small pores [5,76,87]. It has been postulated that there is an apparent inverse correlation between pore size and the necessary time to reconstruct the membrane; however, this has been a non-intuitive perception that is difficult to fully understand. Moreover, comparing the results obtained with StII, LLO, and aerolysin challenged this hypothesis and reinforced the conception that heterogeneity and stability of the pore are more important than size for membrane repair [5].

## 4. Intracellular Signaling Pathways Triggered by APs

PFTs are excellent models for understanding how cells operate in the face of membrane damage. To date, there is little information on the response to cell membrane injury caused by a particular AP and the time necessary for cells to recuperate from membrane damage. Lesions in the membrane of living cells can differ in size and nature; therefore, these characteristics are expected to govern how cells can handle membrane recovery mechanisms or succumb to damage. In general, cells engage plasma membrane repair pathways, reorganize the cytoskeleton, regulate their metabolic status, and trigger stress-associated signaling [76,88,89,90,91]. Unlike electroporation or mechanical scratching-induced damage which is only bordered by lipids, membrane injury by PFTs causes well-defined and more stable borders [4,90,92]. In recent years, research with different bacterial PFTs has shown that, under subtle osmotic stress, cells can recover from membrane damage. Even though the mechanisms involved in cell membrane recovery are still not well understood, it is commonly accepted that Ca^2+^ and K^+^ ions, as well as cellular ATP, are the key players in the process of membrane recovery in response to aggression. In particular, it has been documented that Ca^2+^ ions are necessary for the repair of large cell membrane disturbances, where internal vesicles (for instance, lysosomes and endosomes) contribute with their membranes to rebuild the impaired membrane [93]. In general, membrane repair after attack by bacterial PFTs can take place by (a) membrane patch detachment, (b) PFT internalization through endocytosis, and (c) Ca^2+^-activated annexin-dependent membrane healing [90,94,95]. In contrast, very small pores similar to those elicited by electroporation of some PFTs are rebuilt in a Ca^2+^-independent manner [76,96,97,98,99,100]. So far, very little is known about which of these mechanisms are activated upon the action of APs in cellular membranes. Besides membrane repair mechanisms, PFTs can activate other intracellular pathways aimed to reinforce their toxic action in cells. We next summarize the main intracellular pathways activated upon the APs membrane injury. These processes can lead either to cell survival or cell death and also contribute to the inflammatory response triggered after toxin-induced cell death.

### 4.1. K^+^ Outflow Promotes the Activation of MAPKs

**Figure 2:**
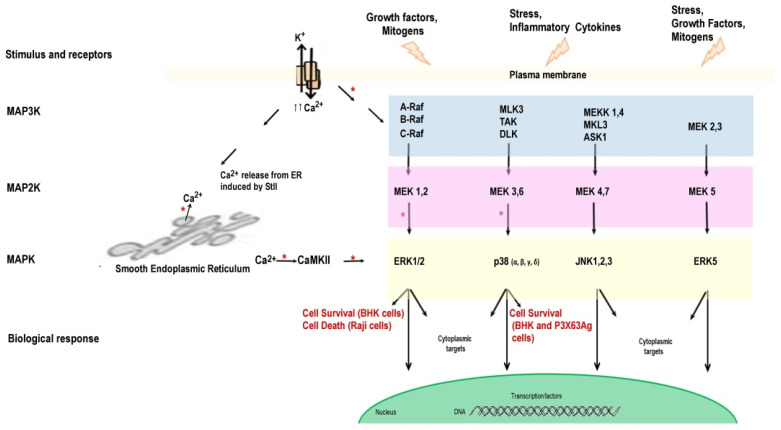
Intracellular MAPK signaling pathways activated under extracellular signaling and StII action. Membrane perturbation induced by various stimuli provokes the activation of the MAPKs cascade. The targets of MAPKs in the cytosol and nucleus are responsible for the biological response leading to cell survival or death. Pore formation by StII induces ERK1/2 activation through MEK1/2 pathway. In normal BHK cells, the inhibition of ERK1/2 increases cell death, suggesting their involvement in cellular defense [5]. However, the ERK1/2 activation in tumor Raji cells is related to cellular death [113]. Pore formation by StII causes K^+^ efflux through the StII’s pore and an increase in intracellular Ca^2+^ from the external medium and ER [113]. As a result, MAP3K activation takes place, eliciting p38 phosphorylation. The activation of p38 kinase is associated with cell survival in BHK [5] and P3X63Ag cells (unpublished results). * Indicates those processes in which the intervention of StII has been identified.

It has been well documented that cellular K^+^ is a key regulator of the defense response generated in the face of PFTs aggression [6,76,101,102]. Indeed, K^+^ outflow through the pores triggers downstream signaling cascades that promote cell survival. Invaded cells respond to non-lytic concentrations of PFTs through conserved intracellular signaling pathways, comprising activation of the mitogen-activated protein kinases (MAPKs), critical to cell survival [103,104]. MAPKs pathways can be clustered into four main subsets: the extracellular signal-regulated kinase 1 and 2 (ERK1/2) pathway (also recognized as the classical pathway), the c-Jun N-terminal kinase (JNK) pathway, the p38 pathway, and the ERK5 pathway [105,106,107] (Figure 2). The decrease in intracellular K^+^ due to membrane perforation leads to the activation of one or all of these kinase subsets [77,108]. Such activation leads to the transcriptional regulation of a wide variety of physiological activities implied in the recovery of cell membrane integrity, adaptation, and cell survival [85,86,103,104,109,110,111,112].

However, there was no report on the association between these intracellular events and the activity of APs. To shed light in this regard, we treated BHK cells with StII at sublytic concentrations and assessed the effects on kinase activity by a phospho-array comprising kinase antibodies against 29 dissimilar kinases 20 min after exposure to the toxin in conditions of decreased intracellular K^+^ [5]. The response was very specific and quite similar for StII and LLO, which has been proved to elicit MAPK phosphorylation in target cells [76]. Indeed, our study revealed that StII and LLO promoted the phosphorylation of both p38 and Erk1/2 MAPKs. Additionally, LLO triggered the activation of the cellular transcription factor cAMP Response Element-Binding (CREB), as had been previously demonstrated [76]. It is worth noting that 2 h post-activation, Erk1/2 was dephosphorylated, indicating that activation occurred transiently as a result of StII action [5]. Moreover, Erk1/2 phosphorylation was not observed when cells were pre-treated prior to toxin addition with the MEK1/2 inhibitor U0126 indicating that Erk1/2 activation occurred via its upstream kinase MEK1, as it has been documented for other bacterial toxins [76]. Furthermore, the relevance of p38 and Erk1/2 kinase activation in restoring K^+^ balance after membrane injury by StII was demonstrated. Indeed, when cells were pre-incubated with either MEK- (U0126) or p38 inhibitor (SB203580), no changes in the kinetics of StII-induced K^+^ outflow were observed; however, it significantly impaired recovery of intracellular K^+^ [5]. Remarkably, ion traffic might not be exclusively intermediated by the pores themselves [114]; as for APs, activation of endogenous K^+^ channels has been claimed to participate as a regulatory mechanism of StII-induced cell swelling [72].

StII activity on cells provokes swelling and membrane blebbing [5]. Blebs have been identified in animal cells attacked by other PFTs such as EqTII [74], streptolysin O, from *Staphylococcus aureus*, and parasporin-2, from *Bacillus thuringiensis* [79,115]. Although the function of blebbing under these circumstances is still contentious, it has been argued that it could function as a reservoir for the augmented cellular volume of water, probably having a defensive role against cell lysis [78,79,80]. Therefore, the bleb’s appearance supports the conception that eukaryotic cells can protect themselves against AP insult at least in response to StII [5] and EqTII damage [74]. However, these repairing mechanisms do not appear to be effective at elevated toxin concentrations, at which the cell cannot recover and finally dies by necrosis [4,60,116]. Our study provided evidence that StII is capable of stimulating effective mechanisms for the recovery of the cell membranes integrity in an analogous way to the large-pore-forming toxin LLO [5]. To date, there has been no other report of a PFT that forming small pores in the membrane is capable of inducing rapid plasma membrane recovery [5]. At least in BHK cells, kinases p38 and Erk1/2, in contrast to JNK, are the major kinases involved in the defense mechanisms against StII aggression. This selective involvement of two kinases subsets indicates that some but not all pathways are equivalently important in response to different toxins. Additionally, it must be considered that a given cell line may respond in a particular way to the injury provoked by a PFT. As a result of the activation of these MAPKs, a complex cascade signaling mechanism and various defense processes are triggered [5] (Figure 2).

Briefly, we showed that the reduction of intracellular K^+^ appeared immediately in BHK cells as a consequence of StII treatment. However, restoration of physiological K^+^ levels and survival of BHK cells were dependent on StII concentration. When cells were assaulted with a high concentration of StII, the damage caused to the plasma membrane was irreparable and led to cell death. In contrast, at low concentrations of the AP, K^+^ efflux was able to promote the activation of kinases p38 and Erk1/2, which was relevant to restoring K^+^ balance after StII membrane injury [5]. This is the first study to demonstrate that APs act similarly to bacterial PFTs and induce MAPK pathways, which are involved in many important intracellular physiological processes [5] (Figure 2).

### 4.2. Ca^2+^ Is a Relevant Signaling Mediator of Cell Death

The pores formed by AP EqTII cause Ca^2+^ entry into bovine lactotrophs [71] and neuroblastoma cells, leading to a concomitant influx of water and thus cell swelling [117]. Increased intracellular Ca^2+^ concentration can also trigger one or several cascades of Ca^2+^-dependent kinases leading to cell survival or death. Different pathways and kinases are susceptible to changes in the intracellular Ca^2+^ concentration, including the Erk1/2 MAPK pathway, the cAMP-dependent protein kinase A (PKA), the Ca^2+^/calmodulin-dependent protein kinase II (CaMKII), the Ca^2+^/phospholipid-dependent protein kinase (PKC), and the phosphatidylinositol-3-kinase (PI3K) [116].

The action of EqTII or Bc2, a cytolysin produced by the sea anemone *Bunodosoma caissarum* [118], on human glioma U87 cells did not reveal any cellular alterations when visualized by cytochemical cell staining, such as shrinkage or chromatin condensation, characteristic features of apoptotic cell death [85]. Furthermore, no difference in the amount of TUNEL-positive nuclei, a procedure for detecting DNA fragmentation, was observed between treated and untreated U87 cells. Nevertheless, at the same concentration, both toxins induced cell lysis. These results imply that glioma cell viability is compromised by a non-apoptotic necrotic-like cell death mechanism [85]. In addition to describing the general characteristics of cell death prompted by these APs, the cell signaling pathways by which these toxins induced their toxicity on U87 cells were also investigated. For this purpose, cells were preincubated with inhibitors of significant elements of cell death signaling, for instance, the pathways Erk1/2, PKC, PKA, PI3K, and CaMKII. In turn, the CaMKII inhibitor, KN-62, and the inhibitor of MEK1 and MEK2, PD98059, blocked the loss of cell viability promoted by both APs. Moreover, the decrease in cell viability prompted by Bc2 and EqTII resulted completely abrogated by pretreatment with staurosporine, a broad-spectrum PKC inhibitor. On the other hand, PKA and PI3K were not involved in the mechanism of cell death triggered by Bc2 or EqTII. Indeed, cells incubated with the specific PKA inhibitor H89 or with the PI3K inhibitor LY294002 did not affect cell death prompted by Bc2 or EqTII. In summary, this study demonstrated that Bc2 and EqTII induced cytotoxicity in U87 glioblastoma cells with a phenotype similar to necrosis and the involvement of MAPK/Erk, CaMKII, and PKC signaling pathways connected to Ca^2+^ signaling [85].

More recently, we showed that at low concentrations, StII activates the MAPKs Erk1/2 and the Receptor Interacting Protein 1 (RIP1) pathways [60]. In this study, we investigated the cytotoxicity and intracellular responses elicited by StII on human B-cell lymphoma (Raji) in vitro. In contrast to the results obtained for other PFTs [77], we showed that the cell death mechanism provoked by StII in Raji cells occurs without the activation of apoptotic indicators, such as caspases activation, chromatin condensation, DNA fragmentation, or formation of apoptotic bodies indicating that StII induces cell death by a non-apoptotic mechanism in Raji tumor cells. Moreover, the results indicate that StII does not elicit pyroptosis since the pretreatment of cells with the broad-spectrum caspase inhibitor QVD-OPh did not affect the lethal action of StII [60].

On the other hand, StII cytotoxicity requires a functional actin cytoskeleton, induces cell swelling, lysis, and the resulting release of cellular content. Of note, StII induces Ca^2+^ release, primarily from the Endoplasmic Reticulum (ER), and eukaryotic initiation factor 2α (eIF2α) phosphorylation, both events related to ER stress [113]. The phosphorylation of eIF2α seems to be cytoprotective during the stress of the ER because inhibition of the eIF2 alpha translation initiation activity reduces global protein synthesis. To explore the involvement of ER on the cell death mechanism triggered by StII, we investigated the effect of salubrinal, an inhibitor of eIF2α dephosphorylation. [119]. Indeed, salubrinal decreased the Ca^2+^ concentration in the cytosol, indicating the implication of ER in this process. Because StII elicited eIF2α phosphorylation and given that inhibition of eIF2α dephosphorylation by salubrinal reduces Ca^2+^ in the cytosol, we are tempted to speculate that the toxin–cell interaction might generate stress signals to ER.

**Figure 3:**
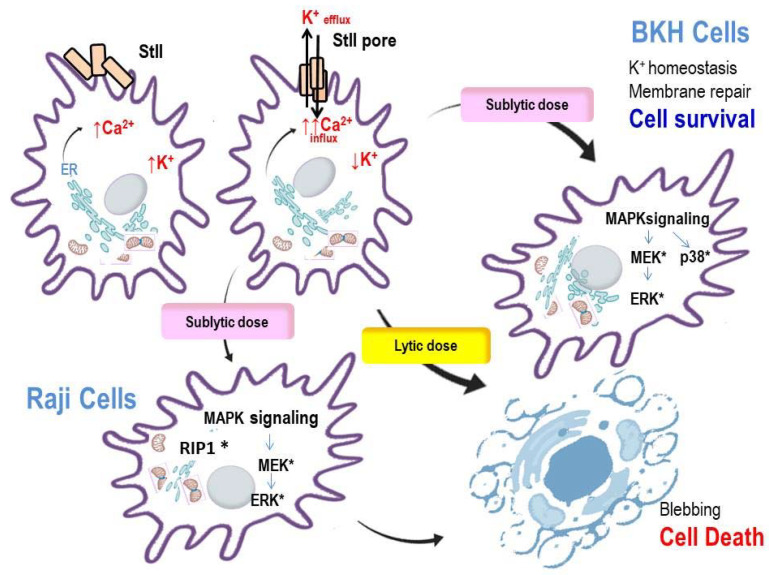
Schematic summary of the main signaling events triggered by StII on normal BHK cells and tumor Raji cells. Upon binding to the membrane, StII promotes Ca^2+^ release to the cytosol from the endoplasmic reticulum (ER) [60]; after pore organization in the membrane a decrease of K^+^ and increase of Ca^2+^ ions occurs in the cytosol. Sublytic doses of StII elicit activation of Erk1/2 and p38 MAPKs, leading to cell survival in BHK cells. This activation occurs in response to K^+^ depletion in cell cytosol [5]. In Raji cells, sublytic doses of StII activate the signaling pathway involving Erk1/2 and RIP1. This activation leads to cell death by a necrosis cell type of cell death with the participation of intracellular pathways. Lytic doses of StII provoke cell necrosis accompanied by blebbing. *: activated components.

In addition to the well-documented involvement of K^+^ in MAPKs activation and membrane restoration, it was shown that in EqTII-induced cell death, Ca^2+^/CaMKII, MAPK Erk1/2, and PKC are involved [85]; remarkably, members of the CaMK cascade are implicated in Erk1/2 activation [116]. In our study, we also found that the treatment of Raji cells with non-lytic concentrations of StII (<1 nM) was enough to activate the Erk constituent of the MAPKs pathway, providing a mechanism by which this toxin can modulate and regulate the cell fate. Notably, the MEK inhibitor PD98059, as well as KN62, a Ca^2+^/CaMKII inhibitor, decreased cell death, indicating that these processes are implied in the mechanism of StII-induced cell death at least in tumor Raji cells [60] (Figure 3). Studies with α-toxin (from *Clostridium septicum*) on Vero cells [112] and with the APs Bc2 and EqTII on neuroblastoma cells [85] also revealed the contribution of the MAPKs pathway to the cytotoxicity of these toxins. In contrast, we have previously shown that StII induced activation of the Erk1/2 pathway was associated with membrane reparation and survival in non-tumor BHK cells [5]. These apparently contradictory results emphasize the notion that MAPK activation and the ultimate fate of the cell, survival or death, will depend on the particular toxin, the cell type, and its metabolic status, stressing the plasticity of the cellular response and questioning any a priori generalization [6]. In any context, Erk1/2 activation seems to be a downstream consequence of membrane perturbation induced by StII. Furthermore, it has been demonstrated that activation of Erk1/2 correlates with the rise in intracellular Ca^2+^, stressing that the Ca^2+^ signal slightly anticipates the activation of MAPKs, consistent with the function of this cation as an upstream activator of MAPKs [120]. Upon stimulation, Erks (Erk1/2/3) are phosphorylated and released from the cytoplasmic compartment to allow their translocation to the nucleus. Remarkably, Ca^2+^ has recently emerged as a regulator of the Erks localization in cells. Indeed, an elevated Ca^2+^ concentration in the cytosol prevents the translocation of activated Erks from the cell cytosol to the nucleus by precluding its passage through the nuclear envelope. By this means, Ca^2+^ appears as a regulator of the interaction of Erks with relevant substrates, and hence their signaling specificity [121]. One can hypothesize that a fine tuning between the phosphorylation level of Erks and the availability of Ca^2+^ in the cytosol could be the reason for the ultimate cell fate under APs attack in normal or tumor cells (Figure 3).

### 4.3. Signaling via the Pattern Recognition Receptors

StII creates membrane pores that alter cytosolic ion composition triggering diverse intracellular MAPKs pathways. Their ultimate impact on cell survival or death will depend on toxin concentration and—probably—the diverse intracellular metabolic environment of the tumor [60] or non-tumor [5] cell phenotype. However, we still do not have enough robust experimental evidence to draw a precise picture correlating the activation of a given pathway with the fate of a particular cell type.

Many pathogenic bacteria activate the host’s innate immune system by secreting PFTs that deplete the cytosol K^+^ concentrations of the host. In fact, it has been demonstrated that reduction of K^+^ concentration in cytosol is a key factor for the NLRP3 inflammasome activation compared to other stimuli [84,122]. However, there is no evidence whether the depletion of cytosolic K^+^ caused by StII [5,75,76] can also stimulate the NLRP3 inflammasome activation as it occurs with other PFTs from bacteria [84,122]. It is an interesting question that still remains open and deserves our attention due to the possible applications of Sts.

In addition to MAPKs and the NLRP3 inflammasome, a third important signaling pathway involves pattern recognition receptors (PRRs) which are commonly found on the cell membrane, endosomal membrane, or cytoplasm [123]. PRRs include the Toll-like receptors (TLR), the C-type lectin receptors, the Nod-like receptors, the AIM2-like receptors, intracellular DNA sensors such as cGAS as well as retinoic acid-inducible gene-I-like receptors [124]. Each PRR binds to its ligands on the plasma or endosomal membrane and in the cell cytosol, or to proteins of the respective signaling pathway. The lipopolysaccharide (LPS) of Gram-negative bacteria is recognized by TLR4, and this recognition triggers activation of the host’s innate immune system. The binding of TLR4 to LPS leads to the dimerization of TLR4 via its cytoplasmic Toll/IL-1 Receptor (TIR) domain. In turn, the TIR domain is associated with TIR-domain-containing adaptor proteins such as the adaptors MyD88 and TRIF, the TIR-domain-containing adapter-inducing interferon-β, activating a number of downstream pathways leading to the expression of Nuclear Factor Kappa B and other transcription factors that promote inflammation [125].

So far, there is no evidence of direct recognition of PRR by APs; nonetheless, we cannot dismiss the possibility that these PFTs may trigger downstream signaling pathways via PRR. We recently described the major role of the macrophage vacuolar pathway concerning the antigen cross-presentation elicited by StII encapsulated into liposomes, which is a key process related to the response of CD8+ cytotoxic T lymphocytes [126]. This study is one of the components of a larger project aiming to develop a vaccine platform composed of Sts and liposomes [7,8]. Remarkably, StII has the capacity to maturate dendritic cells [8] in a TLR4/MyD88-dependent way, which contributes to the response of CD8+ cytotoxic T lymphocytes produced by StII-containing lipid vesicles [127]. Besides phagocytic receptors, others such as TLRs also appear to impact the phagosomal maturation favoring antigen presentation and the induction of an immune response [128,129]. Similarly, macrophage activation by StII might occur and prompt the recruitment of the surface antigen-presenting molecules MHC class I in phagosomes. Additional studies will be aimed at testing this hypothesis.

## 5. Conclusions and Perspectives

Despite the commonly accepted notion that APs only perturb the cell membrane physically by installing a proteo-lipidic pore, the current vision of the APs action can now be expanded. The extent of its action on cells appears to depend on toxin concentration, the cell type, and the presence of the receptor on the target cell membrane. These arguments suggest that APs could generate an innate immune response in a similar way to that elicited by bacterial PFTs. Thus, APs could orchestrate a series of interconnected reactions that could lead to necrosis, apoptosis, pyroptosis, or any other mechanism of cell death and, of course, cell survival. As it is known, all APs show elevated hemolytic activity whose molecular mechanism of cell death appears to be necrosis, as mature mammalian erythrocytes have no nucleus, ER, or mitochondria and are thought to have a limited capacity to undergo apoptosis [130]. Our current perception of the predictable effects of an AP at sublytic concentrations has diversified and provides us new tools for its possible applications in biomedical or nanobiotechnological fields.

Due to their highly cytolytic activity, APs are attractive molecules for the generation of immunotoxins (ITs) targeting the cell membrane. In particular, the APs produced by *S. helianthus* [61,62,63,131] and *A. equina* [132] have been used in the construction of ITs. These AP-derived ITs could, besides their killing activity, boost the effectiveness of diverse common antineoplastic drugs by easing their access to cell cytosol and possibly alleviating their side effects by decreasing the effective drug dose [45]. More recently, sticholysins have emerged as prospective tools for antigen delivery systems to antigen-presenting cells and as the main active component of a platform for vaccine development [7,8].

A more thorough and comprehensive understanding of the activity displayed by APs inside the cell as a result of their association with the membrane will contribute to a more accurate assessment of their possible role in different applications and to an enhanced understanding of their mechanism of action. Similarly to their bacterial counterparts, the biological impact of APs in cells has its initial anchorage point at the plasma membrane, but the effects are much more profound and cross-cut several signaling pathways such as MAPKs, RIP-catalyzed cascade, interaction with TLRs, and would possibly also be involved in the stimulation of the NLRP3 inflammasome. Future research in this field should be supported by the findings summarized here and complemented by new experimental evidence.

## Data Availability

Not applicable.
